# Diagnostic work-up and loss of tuberculosis suspects in Jogjakarta, Indonesia

**DOI:** 10.1186/1471-2458-12-132

**Published:** 2012-02-15

**Authors:** Riris Andono Ahmad, Francine Matthys, Bintari Dwihardiani, Ning Rintiswati, Sake J de Vlas, Yodi Mahendradhata, Patrick van der Stuyft

**Affiliations:** 1 Centre for Tropical Medicine, Faculty of Medicine, Gadjah Mada University, Jogjakarta, Indonesia; 2 Department of Public Health, Erasmus MC, University Medical Centre Rotterdam, Rotterdam, The Netherlands; 3 Unit of Epidemiology & Disease Control, Department of Public Health, Institute of Tropical Medicine, Antwerp, Belgium; 4 Department of Microbiology, Faculty of Medicine, Gadjah Mada University, Jogjakarta, Indonesia

**Keywords:** Diagnostic work-up, Tuberculosis, TB DOTS facilities, Indonesia

## Abstract

**Background:**

Early and accurate diagnosis of pulmonary tuberculosis (TB) is critical for successful TB control. To assist in the diagnosis of smear-negative pulmonary TB, the World Health Organisation (WHO) recommends the use of a diagnostic algorithm. Our study evaluated the implementation of the national tuberculosis programme's diagnostic algorithm in routine health care settings in Jogjakarta, Indonesia. The diagnostic algorithm is based on the WHO TB diagnostic algorithm, which had already been implemented in the health facilities.

**Methods:**

We prospectively documented the diagnostic work-up of all new tuberculosis suspects until a diagnosis was reached. We used clinical audit forms to record each step chronologically. Data on the patient's gender, age, symptoms, examinations (types, dates, and results), and final diagnosis were collected.

**Results:**

Information was recorded for 754 TB suspects; 43.5% of whom were lost during the diagnostic work-up in health centres, 0% in lung clinics. Among the TB suspects who completed diagnostic work-ups, 51.1% and 100.0% were diagnosed without following the national TB diagnostic algorithm in health centres and lung clinics, respectively. However, the work-up in the health centres and lung clinics generally conformed to international standards for tuberculosis care (ISTC). Diagnostic delays were significantly longer in health centres compared to lung clinics.

**Conclusions:**

The high rate of patients lost in health centres needs to be addressed through the implementation of TB suspect tracing and better programme supervision. The national TB algorithm needs to be revised and differentiated according to the level of care.

## Background

Early and accurate diagnosis of pulmonary tuberculosis (TB) is critical for better treatment outcomes and reduced transmission [1,2]. The gold standard for TB diagnosis is bacilli culture in Löwenstein-Jensen (LJ) media, but this is difficult in many resource-poor settings. In addition, LJ culture takes 6-8 weeks, which limits its usefulness as a first-line diagnostic test. Rapid cultures in liquid media provide faster results but are more expensive and prone to contamination [3]. Therefore, the World Health Organisation (WHO) and the International Union Against Tuberculosis and Lung Disease (IUATLD) endorse sputum smear microscopy with at least one positive sputum smear for a diagnosis of smear-positive TB [4,5]. For patients with suspected TB and negative sputum results, the diagnostic criteria for smear-negative pulmonary TB encompasses at least two negative smears, radiographic abnormalities consistent with active pulmonary TB, no response to a course of broad-spectrum antibiotics, HIV-positive status, and a decision by a clinician to treat with a full course of anti-TB chemotherapy [4].

The WHO recommends the use of diagnostic algorithms to diagnose smear-negative pulmonary TB [6], and many TB-endemic countries have adopted an algorithm approach [7]. However, the effectiveness of diagnostic algorithms is influenced by local factors, such as HIV prevalence, adoption of the algorithm at frontline health care services, and the adherence of local clinicians to the algorithm [8]. Several studies have evaluated diagnostic algorithms, mainly focusing on the performance of the algorithm in diagnosing smear-negative TB among HIV patients [8,9] or in specific settings [10-13]. However, studies that evaluate the implementation and adherence to such algorithms in routine settings are lacking, though they would help programme managers identify and correct possible weaknesses in the diagnostic process. The present study evaluated the implementation of the national TB diagnostic algorithm, particularly health providers' adherence to the standardised diagnostic algorithm, in routine health care settings in Indonesia. The national TB diagnostic algorithm was adopted from the WHO treatment guidelines, which are designed particularly for application in health centres. The diagnostic algorithm was implemented at the investigated facilities, and most of the medical staff received training and were familiar with the use of this algorithm.

## Methods

### Study setting

Jogjakarta municipality is an urban area with a population of approximately half a million. The TB control programme in Jogjakarta municipality relies on a network of 18 public health centres, 2 lung clinics, and 9 public and private hospitals. The health centres consist of four microscopy health centres (MHC) capable of performing smear microscopy and 14 satellite health centres (SHC) that collect sputum specimens from TB suspects, prepare smear slides, and send the slides to the MHC for microscopy. The lung clinics have smear microscopy, chest radiography, and VCT services. The incidence of TB in Jogjakarta municipality is estimated to be 63/100,000 [14], and the HIV prevalence among TB patients is estimated to be 1.9% [15]. The TB diagnosis network is supported by an external quality assurance mechanism through quarterly cross-checks by the Provincial Health Laboratory, with technical assistance from the Microbiology Laboratory, Faculty of Medicine, Gadjah Mada University (FM GMU).

### Data collection and analysis

The study took place between November 2009 and May 2010 in all of the public health centres and lung clinics in Jogjakarta municipality. All TB suspects registered at the health centres and lung clinics were included. The definition of TB suspect from the national TB control programme (NTP) guidelines was applied; a patient with a cough for more than 2 weeks was considered a TB suspect, irrespective of HIV status [16]. Diagnostic work-ups, defined as clinical and laboratory examinations, were documented prospectively. The diagnostic work-up started at the time a patient was considered a TB suspect and continued until the time of diagnosis. A clinical audit form used to record the diagnostic work-up chronologically, step-by-step was developed and tested prior to data collection. Data on the patient's gender, age, symptoms, examinations (types, dates, sequence, and results), and final diagnosis were collected. Patients who did not return to their previous health facility, even if they completed the diagnostic work-up, were considered lost and not traced back. A trained nurse filled out the audit form in each facility. A field coordinator conducted monthly supervision visits to check the completeness of the collected data and cross-check it with the patients' clinical files and the laboratory register.

**Figure 1:**
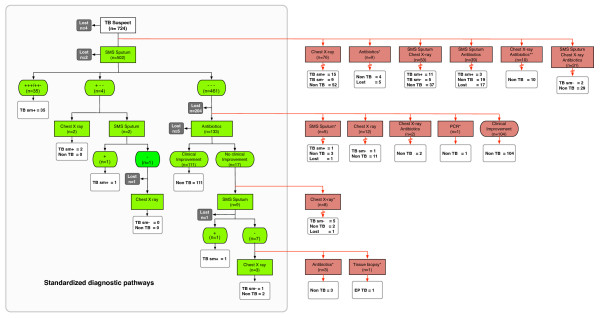
**Pathway of diagnostic work-up of patients suspected of having TB in lung clinics and health centres**. Green boxes represent the standardised national TB diagnostic algorithm and the number of patients whose diagnostic work-ups followed the standardised algorithm. Red arrows and boxes represent diagnostic work-ups that did not follow the standardised algorithm. Black boxes represent patients who were lost during diagnostic work-up. Broken arrows represent subsequent diagnostic examinations not shown in the graph. All patients who completed diagnostic work-up underwent sputum smear examinations at a certain point during their diagnostic work-up. After each sputum examination, or series of sputum examinations, the possible outcomes are indicated by '+' (positive) or '-'(negative). TB: tuberculosis; EP TB: extra-pulmonary TB; TB sm+: smear-positive tuberculosis; TB sm-: smear-negative tuberculosis. * Pathway that occurred in health centres only. ** Pathway that occurred in lung clinics only

We compared the actual diagnostic work-up with the national TB diagnostic algorithm (Figure 1). All cases that did not match the standardised pathways were categorised as other pathways.

All data were double entered into a database checked for typing errors, missing data, and inconsistencies. Data were analysed using SPSS for Windows version 16. Logistic regression was used to analyse factors associated with patient loss, positivity of TB diagnosis among suspects who completed the diagnostic work-up, and smear positivity of TB cases. Factors were first tested univariately. Factors with P < 0.20 were maintained using a backward Likelihood Ratio (LR) approach in the multivariate model. Chi-square was used to assess the background characteristics of patients visiting lung clinics and health centres. A median-based test was used to assess differences in the diagnostic delay between lung clinics, MHC, and SHC.

Approval was obtained from the ethical committee of FM GMU, Indonesia and the ethics committee of the University of Antwerp, Belgium.

## Results

**Figure 2:**
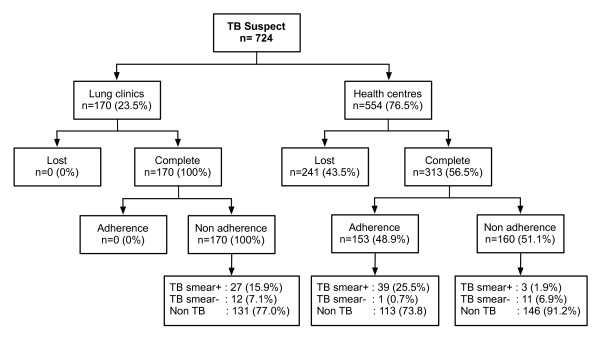
**Adherence to the national TB diagnostic algorithm and outcomes of diagnostic work-ups in lung clinics and health centres**.

A total of 724 patients were registered as TB suspects during the study. The majority, 76.5% (n = 554), attended health centres and 23.5% (n = 170) attended lung clinics (Figure 2). The gender and age distribution of patients consulting at health centres and lung clinics was similar (data not shown). Of patients who consulted health centres, 43.5% (n = 241) were lost, but none from the lung clinics were lost. Among patients who completed the diagnostic work-up, adherence to the national TB diagnostic algorithm was 0.0% in lung clinics and 48.9% (n = 153) in health centres.

Figure 1 shows a summary of the diagnostic pathways in lung clinics and health centres. Most of the patients (n = 502, 69.7%) started their diagnostic work-up with microscopic sputum smear examination, but only 21.1% (n = 153) followed the standardised algorithm and completed the diagnostic work-up.

Of 461 suspects who first received sputum smear examinations but had negative results, 136 (29.5%) followed non-standardised pathways (Figure 1) and 22.6% (n = 104) were diagnosed as non-TB because of clinical improvement. Approximately 30% (n = 218) of patients did not follow the national algorithm from the start of the diagnostic work-up. The diagnostic work-up started with chest radiography, an antibiotic trial, or a combination of these procedures with sputum smear microscopy. Notably, all TB suspects who were not lost to follow up underwent sputum smear microscopy at some point during their diagnostic work-up.

Patients were lost at every level of the diagnostic work-up (Figure 1), even at very early stages. The highest frequency of loss occurred after an initial set of negative sputum results, 44% (n = 204) of the 461 smear-negative patients. Patient loss was similar in males and females but varied slightly among different age groups (Table 1). Patient loss was significantly higher in SHC compared to MHC (OR 2.06, 95% CI 1.41-3.02, P < 0.001).

**Table 1:** Determinants of patient loss among 554 TB suspects in health centres in Jogjakarta municipality

Variable	n/N	%	OR	Univariate 95% CI	P-value
All	241/554	43.3			
First step work-up					
Sputum smear microscopy	215/497	43.3	1		
Other ^Ť^	22/53	41.5	0.93	0.52-1.65	0.81
Nothing ±	4/4	100.0			
Sex					
Male	115/262	43.9	1.03	0.74-1.44	0.86
Female	126/292	43.2	1		
Age group (years)					0.10
18-25	28/81	34.6	0.70	0.39-126	0.23
26-35	40/88	45.5	1.10	0.62-1.94	0.74
36-45	47/89	52.8	1.48	0.84-2.59	0.18
46-55	45/92	48.9	1.26	0.72-2.21	0.41
56-65	34/95	35.8	0.74	0.42-1.29	0.29
> 65	47/109	43.1	1		
Type of health facility					
MHC	53/168	31.5	1		
SHC	188/386	48.7	2.06	1.41-3.02	< 0.001*

^Ť ^Chest X-ray, antibiotics, or combination of X-ray and antibiotics with/without sputum smears

^±^Suspects who were directly lost before any examination was performed. Not included in the statistical analysis

*Only this variable remained in multivariate analysis

For the TB suspects who completed the diagnostic process, the type of health facility and gender was not associated with TB diagnosis (data not shown); only older age was associated with TB diagnosis (OR 0.75, 95% CI 0.66-0.86, P < 001). No significant differences were found between patients diagnosed with smear-positive TB and smear-negative TB in terms of gender, age group, and type of health facility where they were diagnosed (data not shown), and only sputum smear as the first examination was associated with TB-positive smears (OR 2.93, 95% CI 1.10-7.77, P < 0.03).

The median duration of diagnostic delay for smear-positive cases, excluding the patients who dropped out, was 1 day in lung clinics, 4 days in MHC, and 7.5 days in SHC. The median delay until diagnosis for smear-negative TB and non-TB cases was also longer in the SHC compared to MHC and lung clinics. The median tests showed significantly longer diagnostic delays in SHC and MHC compared to lung clinics among smear-positive, smear-negative, and non-TB patients (Table 2).

**Table 2:** Duration of diagnostic delay (in days) according to the diagnostic outcome for TB suspects who completed the diagnostic work-up

		Lung clinics		MHC		SHC	P-value
	n	Median (IQR)	n	Median (IQR)	n	Median (IQR)	
TB smear-positive	27	1 (1 - 1)	16	4 (2 - 7)	26	7.5 (5 - 12)	< 0.001
TB smear-negative	12	2 (1 - 4)	4	10 (5 - 88)	8	13.5 (5 - 14)	0.006
Non-TB	131	2 (1 - 4)	95	8 (5 - 15)	164	11 (8 - 11)	< 0.001

IQR = inter-quartile range

## Discussion

This study is one of a few that focus on the process of diagnostic work-ups in DOTS facilities. Our study found that all of the diagnostic work-ups in lung clinics and the majority of diagnostic work-ups in health centres did not comply with the standardised national algorithm. No patients were lost in lung clinics, and 43.5% of patients were lost in health centres. The duration of diagnostic delay was longer in health centres compared to lung clinics.

Some limitations of our study need to be taken into account. First, we limited ourselves to the lung clinics and health centres; TB work-ups at DOTS-affiliated hospitals were not documented. However, the hospitals' diagnostic infrastructure and technical capacity are similar to that of the lung clinics, and we expect the variation in diagnostic pathways in these hospitals to be comparable to that of the lung clinics. Second, patients who were lost were not traced back, which reflects the actual performance of the health services. Third, culture examination was not performed because we did not aim to evaluate the effectiveness of the diagnostic algorithm.

Non-adherence to the standardised diagnostic algorithm was high. One important reason for non-adherence to the diagnostic algorithm was a non-TB diagnosis among patients with an initial negative sputum smear who showed clinical improvement. This decision is reasonable clinically, but the existing algorithm does not accommodate this option. If the decision is considered acceptable, non-adherence in the health centres drops to 18.2% (n = 57). Studies in Ethiopia and India have also shown a high rate of non-adherence [17-19]. Though different types of non-adherence exist (e.g., sequence of examination, inadequate examination, or absence of recommended examination [17-19]), we found that the sequence of examination was the main reason for non-adherence. Most patients in lung clinics, in particular, received multiple examinations at the first consultation due to the availability of chest radiography. Also, specialised chest doctors are more likely to rely on their clinical judgment than on the diagnostic algorithm.

The proportion of patients lost in health centres was surprisingly high. A study in Pakistan reported a lower proportion of lost patients (13%), but 5.2% of these patients were smear-positive cases and, therefore, at high risk of spreading infection [20]. We found that gender did not correlate with the default rate, whereas another study found that males defaulted more frequently than females [20]. Patient loss in SHC was twice as high as loss in MHC. Several studies have suggested that the quality of services is a common reason for defaulting before initiating treatment [20-22]. One possible explanation in our study could be the substantially longer duration of the diagnostic work-up or inadequate information provided to the patients in SHC. A study in a similar setting in Java indicated that only 20% of the nurses in health centres provide adequate information to TB suspects regarding sputum samples [23]. Because patients were not traced, we do not know if they felt better or consulted other health services. As the subsequent consultations, if any, were all self-referral, we did not consider the subsequent consultation as part of the TB diagnostic work-up under study. A possibility of self-selection (i.e. patients with minor symptoms went to the SHC, whereas more severe patients preferred to go to the MHC or lung clinics) exists, and the latter group may be more motivated to adhere to health providers' orders.

The diagnostic delays were significantly longer in SHC and MHC compared to lung clinics, most likely due to the diagnostic capacity of the centre, which influences the timing of different examinations. All facilities and skills required to perform a smear-negative TB diagnosis are readily available in lung clinics, whereas SHC need to send the sputum smears to the referral MHC and wait for the report of the results. Also, in the case of chest radiography by the health centres, most patients need to be referred to higher-level hospitals or lung clinics, which causes further delay. Furthermore, lung clinics tend to perform multiple examinations at the same contact. Although this practice is not in line with the national diagnostic algorithm, it significantly reduces the duration of a diagnostic work-up.

All diagnoses at the lung clinics were made without complying with the national diagnostic algorithm, but this considerably reduced the diagnostic delay and prevented patient loss. Getahun et al. argued that the health service delay introduced by applying the TB diagnostic algorithm in a linear fashion could be life threatening, particularly among HIV-positive patients, as it needs 11-34 days to establish the diagnosis of smear-negative pulmonary TB under the most optimistic scenarios [7]. An additional guideline to expedite the TB diagnosis process was published specifically for HIV-positive smear-negative TB suspects [6]. The Stop TB Partnership has endorsed an international standard of tuberculosis care (ISTC) since 2006 [24]. The standardised diagnostic algorithms are developed to improve the sensitivity and specificity of TB diagnosis among health providers within the TB control programme, particularly in primary health centres, whereas the purpose of the ISTC is to provide guidelines that are more suitable for all practitioners, particularly those working in the private sector, and allow some degree of flexibility. The ISTC recommends sputum smear examination of all patients suspected of having pulmonary TB and all persons with chest radiographic findings suggestive of TB. The ISTC also acknowledges that many providers do not adhere to the TB diagnostic algorithm in a sequential fashion, and that the approach outlined in the algorithm may be quite costly to the patient as it may take several visits to complete the diagnostic work-up, deterring her/him from continuing with the diagnostic evaluation. Therefore, the ISTC recommends that the application of such an algorithm be done in a flexible manner [25]. Our study found that all TB patients who completed the diagnostic work-up in lung clinics or health centres underwent sputum examination at some point during their diagnostic work-up, which still conforms to the ISTC recommendations. Considering the diagnostic capacity of lung clinics, applying the ISTC is more suitable than following the standardised national TB diagnostic algorithm. A recent study also suggested that the performance of the WHO-based diagnostic algorithm for HIV-negative TB suspects is far from optimal [12]. Another study among HIV-positive patients also suggested that chest radiography is the best next step after sputum examination [9]. Clearly, the Indonesia national TB programme (NTP) needs to re-evaluate the current diagnostic algorithm and policy, taking into account possible scenarios at different levels of care, new evidence, and recent international policy recommendations.

The high rate of patients lost in health centres requires special attention. Improving service quality through better communication with patients may be the answer. However, Khan et al. reported that, even when patients were counselled, the rate of patient loss during diagnostic work-up was not reduced [20]. Faster diagnostic technology may be a better answer. The WHO recently endorsed a new rapid molecular test for TB diagnosis following a successful evaluation in low-income countries [26,27]. The test provides a sensitivity comparable to culture but with results in less than 2 hours. However, considerable resources need to be invested and much time will elapse before this new method will be generally implemented in Indonesia. Therefore, improving the quality of service, implementing patient tracing, offering a diversified national algorithm, and ensuring better programme supervision may be the best options at the moment.

## Conclusions

We conclude that the implementation of the diagnostic work-ups in lung clinics, and to some degree in health centres, does not conform to the national TB diagnostic algorithm, but it does conform to the ISTC. A need exists to offer a diversified national TB algorithm taking into account different levels of care. The high rate of patient loss in health centres needs to be addressed by improving the quality of service and implementing patient tracing and better programme supervision.

## Competing interests

The authors declare that they have no competing interests.

## Authors' contributions

Conception and design of the study: RAA, FM, BD, NR, YM, PVdS. Acquisition of data: RAA, FM, BD, NR, YM. Analysis and interpretation of data: RAA, FM, BD, SJdV, PVdS. Drafting the paper: RAA, FM, SJdV. Substantially revising the paper: RAA, FM, BD, NR, SJdV, YM, PVdS. All authors read and approved the final manuscript

## Pre-publication history

The pre-publication history for this paper can be accessed here:

http://www.biomedcentral.com/1471-2458/12/132/prepub
